# When Onco-Immunotherapy Meets Cold Atmospheric Plasma: Implications on CAR-T Therapies

**DOI:** 10.3389/fonc.2022.837995

**Published:** 2022-02-23

**Authors:** Xiaofeng Dai, Jitian Li, Yiming Chen, Kostya (Ken) Ostrikov

**Affiliations:** ^1^Wuxi School of Medicine, Jiangnan University, Wuxi, China; ^2^CAPsoul Biotechnology Company, Ltd, Beijing, China; ^3^Henan Luoyang Orthopedic Hospital (Henan Provincial Orthopedic Hospital)/Henan Provincial Orthopedic Institute, Zhengzhou, China; ^4^School of Chemistry and Physics and Centre for Biomedical Technologies, Queensland University of Technology, Brisbane, QLD, Australia

**Keywords:** cold atmospheric plasma, onco-immunotherapy, chimeric antigen receptor, off-tumor effect, solid tumor

## Abstract

T cells engineered with chimeric antigen receptors (CAR) have demonstrated its widespread efficacy as a targeted immunotherapeutic modality. Yet, concerns on its specificity, efficacy and generalization prevented it from being established into a first-line approach against cancers. By reviewing challenges limiting its clinical application, ongoing efforts trying to resolve them, and opportunities that emerging oncotherapeutic modalities may bring to temper these challenges, we conclude that careful CAR design should be done to avoid the off-tumor effect, enhance the efficacy of solid tumor treatment, improve product comparability, and resolve problems such as differential efficacies of co-stimulatory molecules, cytokine storm, tumor lysis syndrome, myelosuppression and severe hepatotoxicity. As a promising solution, we propose potential synergies between CAR-T therapies and cold atmospheric plasma, an emerging onco-therapeutic strategy relying on reactive species, towards improved therapeutic efficacies and enhanced safety that deserve extensive investigations.

## 1 Introduction

**Figure 1:**
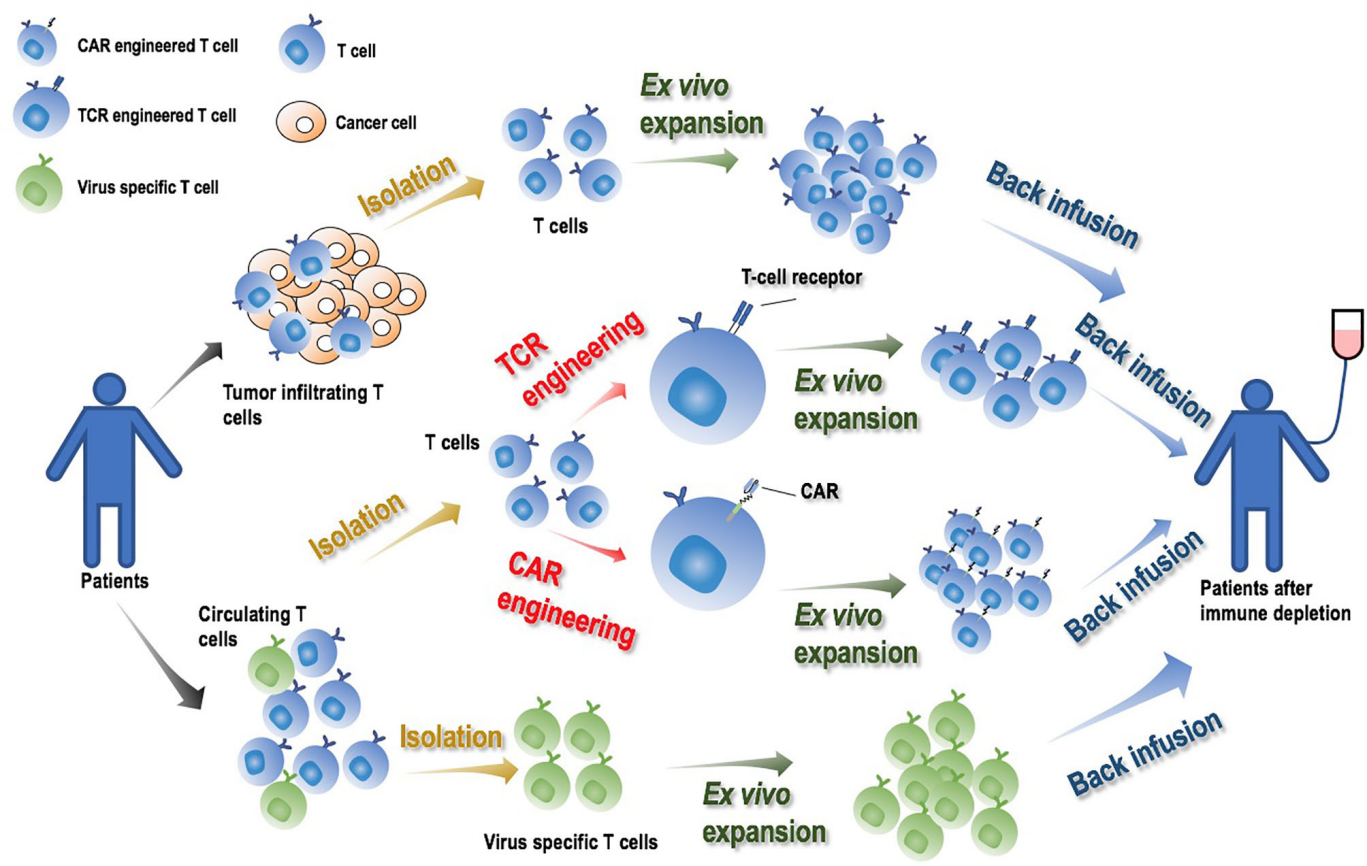
Illustrative diagram on adoptive cell therapy (ACT) and its manufacturing process. By transfusing back one’s own genetically engineered T cells or cancer-cognate lymphocytes that identify and attack cancer cells or foreign invasions, ACT activates the immune system and achieves its onco-therapeutic role. Engineered T cells expressing TCRs or CARs, TILs and virus specific T cells expanded *ex vivo*, are typically used to produce ACT. The manufacturing process is comprised of four major steps: ‘T cell isolation’, ‘T cell engineering’ (for TCR or CAR only), ‘T cell *ex vivo* expansion’, ‘T cell back infusion’. Adjuvant chemo- or radio- therapies are conventionally applied to deplete patients’ endogenous immune cells prior to ACT infusion to allow infused T cells to flourish.

Adoptive cell therapy (ACT) takes advantages of the immune system by transfusing back one’s own genetically engineered T cells or cancer-cognate lymphocytes that identify and attack malignant cells or foreign invasions (1). Adjuvant chemo- or radio- therapies are conventionally applied before ACT infusion to allow infused T cells to flourish by depleting patients’ endogenous immune cells (1, 2). Four types of immune cells are typically used to confer such clinical features, i.e., engineered peripheral blood T lymphocytes expressing T cell receptors (TCRs) or chimeric antigen receptors recognizing tumors (CARs), tumor infiltrating lymphocytes (TILs) expanded *ex vivo*, and T cells specific to viruses (3, 4) (**Figure 1**).

The efficacies of most ACT strategies are limited to certain types of diseases. While infusion of *ex vivo*-expanded TILs has demonstrated its efficacy in curing refractory metastatic melanoma (5, 6), the success is not at present easily transferable to other cancers due to difficulties in collecting tumor-specific T cells (7–9). Transfusing virus-specific T cells is a standard modality for malignancies or infections related to viruses (10–12). TCR therapy functions by enabling cells with new receptors and the ability to recognize antigens against specific cancers and/or trigger other cells to marsh attack on neoplastic cells (13). TCRs can be cloned from the reactive T cells infiltrating patient tumors (14) or humanized mouse models (15, 16), or through phage display (17). TCR must match the human leukocyte antigen (HLA) immune type of the patient genetically before achieving any functionality. To date, TCR therapies have demonstrated their efficacies in shrinking several types of tumors such as melanoma, synovial sarcoma, and colorectal cancers (18–20).

**Figure 2:**
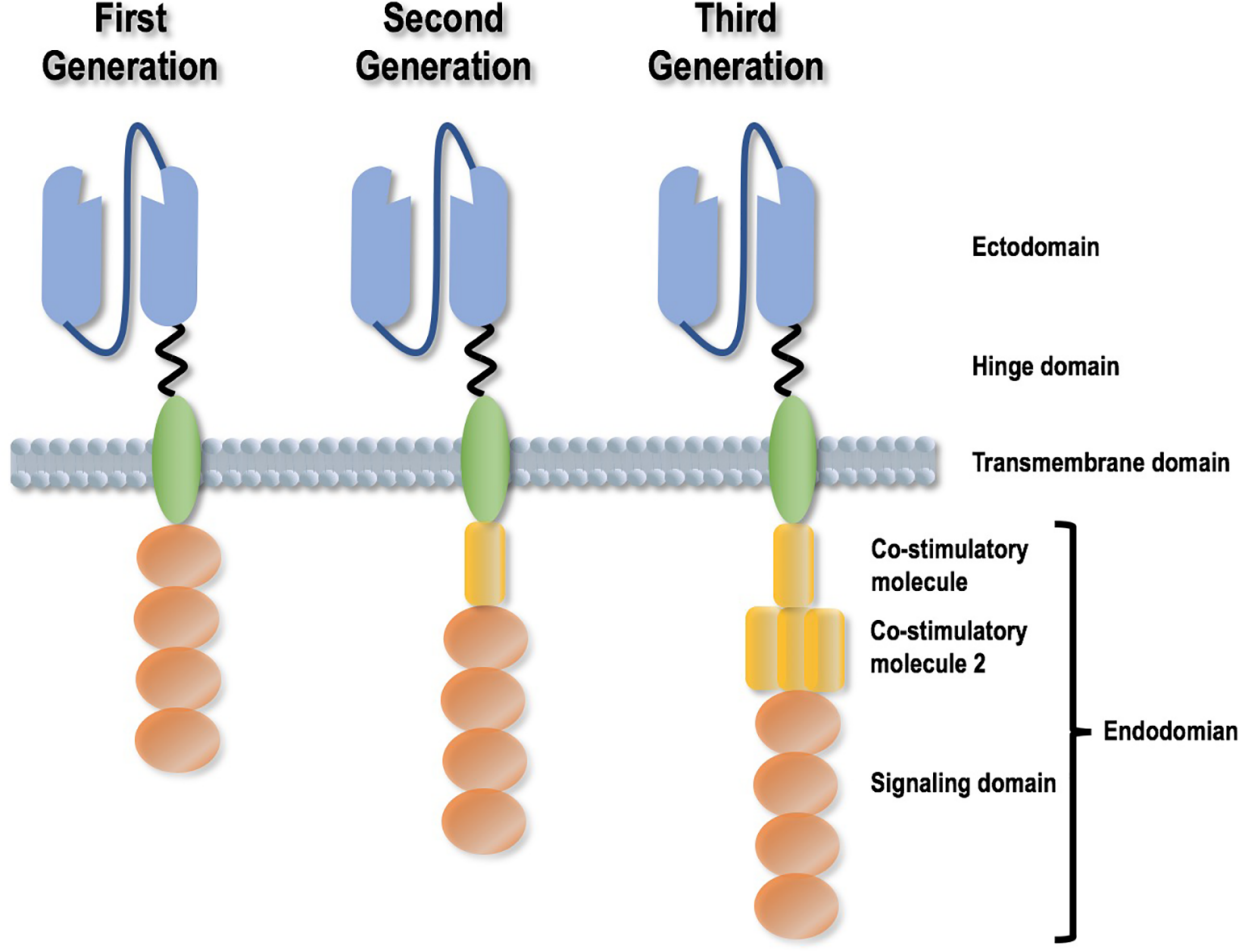
Different generations of Chimeric Antigen Receptor design strategies. A CAR is comprised of four domains, i.e., an ectodomain that is responsible for tumor antigen recognition, an endodomain that contains one or several stimulatory molecules helping T cells persist, a hinge domain, and a transmembrane domain. CAR design has experienced three generations, with the differences reflected by the presence, efficacy and amount of co-stimulatory molecules.

Transducing CAR-T cells to confer specificities on a targeted epitope such as CD19 and BCMA has demonstrated its oncotherapeutic efficacy. CARs are recombinant antigen receptors capable of grafting the specificity of a tumor antigen onto T cells *via* a single chain variable fragment (scFv) derived from an antibody. The aim is to redirect the specificity and function of immune cells and rapidly generate tumor-targeting T cells. A CAR is comprised of four domains, i.e., an ectodomain that is responsible for tumor antigen recognition, an endodomain that contains one or several stimulatory molecules helping T cells persist, a hinge domain, and a transmembrane domain (21) (**Figure 2**). Once expressed on T cell surface, CAR acts as a switch that sets T cells to the attack mode when encountering a matching antigen (22). Any cell surface molecule can be, in principle, targeted by the CAR-T approach, filling in the antigen recognition gaps in the physiological T cell repertoire. Further, CARs do not require antigen processing and presentation and are more broadly applicable to HLA-diverse clinical cohorts. This brilliant idea of transforming T cells into a “living” drug gave birth to the CAR T-cells, whose clinical activity was confirmed in various types of diseases including diffuse large B-cell lymphomas (23). CAR-T cells targeting CD19 have been reported to result in tumor remission of advanced chronic lymphocytic leukemia (CLL) and ALL patients who have failed multiple rounds of chemotherapy (24, 25).

CAR-T therapies have remained as boutique treatments available to a small number of patients despite our complete acknowledgement on their primary roles in rewiring the immune response against tumors. This paper identifies challenges faced by CAR-T therapies limiting their wide applications, reviews ongoing efforts circumventing such problems, and highlights opportunities brought by such promising technologies to academia and clinics (**Table 1**).

**Table 1:** Challenges faced by the CAR-T therapies and the ongoing efforts.

Challenges	Ongoing efforts	References
Off-tumor effect	Double tumor-associated antigen targeting, systematic safety testing of the targeted therapies	(26–30)
Inefficiency in solid tumor treatment	Introduction of chemokine receptors to CAR-T cells, CAR-T cocktail immunotherapy, γδ T cells	(31–36)
Differential efficacies of co-stimulatory molecules	Optimization of ACT combination on a case-by-case basis	(37–39)
Cytokine storm, tumor lysis syndrome	Modulation of co-stimulatory domains, suicide gene design	(40–43)
Myelosuppression, severe hepatotoxicity	Personalization of effective drug duration according to the genetic profile and pathological state of each patient	(44, 45)
Difficulties in product generalization without sacrificing product efficacy	Using off-the-shelf third-party donor gene modified T cells	(46, 47)

## 2 Problems Challenging CAR-T therapies and Possible Solutions

### 2.1 Off-Tumor Effect

One limitation of CAR-T therapy is the requirement of the targets to be solely present on the surface of malignant cells but absent from that of normal cells. The cancer cell antigen epitope needs to be unique for T cell recognition without creating conditions associated with autoimmunity. However, many antigen epitopes found in cancers also have baseline expression levels in normal tissues (48), and targeting an antigen epitope with low specificity may result in severe consequences, namely the ‘on-target off-tumor’ effect, such as EGFR, HER2 (49, 50). For example, a severe transient inflammatory colitis was induced in all three patients (carrying refractory metastatic colorectal tumors) administered with autologous T lymphocytes expressing a murine TCR against human carcinoembryonic antigen (CEA) as CEA is also present in colonic mucosa (51). Off-tumor effects can also be mediated by another protein sharing similar binding affinity with TCR or antigen escape, called the ‘off-target off-tumor’ effect. For example, MAGE-A3 is a cancer-testis antigen expressed in a wide array of malignancies including melanoma (52), cancers of ovary (53), lung (54), bladder (55), colon (56), breast (57), and has been proposed as an immunogenic target in clinics. However, targeting this antigen has resulted in many negative clinical results (52, 58), and even severe consequences (29). The first clinical example of the ‘off-target off-tumor’ effect mediated by TCR targeting MAGE-A3 is Titin (29), which is not related to MAGE-A3 neither structurally nor functionally. In this study, TCR targeting MAGE-A3 caused the death of two patients from heart failure due to the off-target binding to Titin on heart cells (29). In another study, two out of nine cancer patients died after receiving therapies targeting MAGE-A3 in a clinical trial of TCR-engineered T cells; it was found, on further examination, that a family member MAGE-A12 (also possibly, MAGE-A1, MAGE-A8, MAGE-A9) has a low level expression in brain tissue (30). Most top-ranked antigens that could be targeted by CAR-T are also expressed in potentially important normal tissues, such as HER2, EGFR, MUC1, PSMA, and GD2 (10). Although these examples are from failed clinical trials on TCR therapies, they are transferrable to CAR-T therapies and important lessons warranting special care on CAR design. Antigen escape may also result in failure in stimulating immune response. For instance, up to 30% of B-ALL (B cell acute lymphoblastic leukemia) patients receiving CART19 (anti-CD19 CAR-T cells) or blinatumomab (anti-CD19/CD3 antibodies) relapsed due to the loss of CD19 epitope in some tumor cells, imposing a major concern that challenges the efficacy of immunotherapies targeting CD19 (51, 59). Since approval in 2018, tisagenlecleucel (Kymriah, Novartis; Basel, Switzerland) and axicabtagene ciloleucel (Yescarta, Kite Pharma [Gilead]; Santa Monica, USA) are subjected to additional monitoring (CAR T-cell product performance in hematological malignancies before and after marketing authorization). However, the intact immune system in the mice used makes it a less accurate model of human disease than that using immunodeficient mice and thus could not be used to test the safety issues such as the on­target off­tumor activity and cytokine­release syndrome (60).

To avoid or minimize the off-tumor effect, intensive research exploring target candidates with sufficient tumor-specificity and developing novel strategies for therapeutic design have gained traction. The Adaptimmune company has launched extensive safety examination to avoid undesirable therapeutic outcome. The ‘double tumor-associated antigen targeting’ strategy (i.e., targeting two instead of one tumor-associated antigen) has demonstrated its power in generating durable tumor remission; for instance, concomitant expression of CARs targeting CD19/CD20 (28), CD19/CD22 (26), or CD19/CD123 (27) on T cells creates better therapeutic response than pooling T cells carrying either CAR together. However, targeting two tumor-associated antigens may need to reduce the dose of CARs targeting each antigen as the total amount of CAR-T cells could not be increased without limit for the sake of safety; thus, how to appropriately balance the proportion of each CAR expressed on the modulated T cells is of vital importance to create the desired therapeutic efficacy where rigorous computational simulations and experimental validations are needed. It was also suggested that CAR-T cells targeting CD19 induced B precursor acute lymphoblastic leukemia lineage switch towards a more plastic state (61), suggesting the potential of combining therapies targeting cancer stem cells with CART19 or blinatumomab in restoring the sensitivity of cancer cells to anti-CD19 drugs. Despite these experimental efforts, computational approaches, such as establishing databases for target scan and simulating the efficacy of CARs (62, 63), may considerably accelerate this process.

### 2.2 Inefficiency

#### 2.2.1 Inefficiency in Solid Tumor Treatment

The field of CAR-T therapies awaits a clear demonstration of their clinical efficacies in solid tumor treatment, which ultimately determines the validity of this novel modality in the battle against cancer. Though designed to be capable of selectively targeting specific cancer cells, CAR-T cells need to be able to reach the tumor site to take on any effect. While trafficking is not a problem for blood cancers as evidenced by numerous clinical successes (64–67), it is likely to be a bottleneck for delivering therapeutic cells to solid tumors due to the strong immunosuppressive tumor microenvironment. Introducing chemokine receptors such as IL13Rα2 (68), CCR4 (69) and CCR242 to CAR-T cells has been indicated to improve these immune cell trafficking *in vivo* and infiltration into tumors. A recent study reported the feasibility of using CAR-T cocktail immunotherapy, i.e., combined use of CAR-T cells against EGFR and CD133, in the treatment of cholangiocarcinoma, where patients receiving such a therapeutic modality achieved an 8.5- and 4.5-month partial response, respectively (33). Also in 2017, a phase I clinical trial involving 23 metastatic colorectal cancer patients was launched to test the efficacy and safety of a CAR-T product targeting TAG-72 convolving a CD3ζ intracellular signaling domain, and the results showed effective CAR-T cell trafficking to the tumor site and reduced TAG-72 expression without clear evidence of off-tumor toxicity, despite symptoms of anti-CAR immune response in some patients (34). Specific type of immune cells such as γδ T cells have been proposed promising for use in cellular therapy targeting solid tumors (70, 71). Another strategy is to augment the anti-tumor function of CAR-T cells by concomitantly targeting tumor antigens and tumor associated fibroblasts. It is shown that CAR-T cells targeting fibroblast activation protein α together with tumor-associated antigens could considerably enhance the anti-tumor efficacies of CAR-T cells targeting either part alone (36). Also possible is to combine CAR-T therapies with the immune checkpoint inhibitors such as anti-PD-1, anti-CTLA4, OX40, and their combinations such as the joint use of anti-PD-1 and agonist OX40 to create a favorable microenvironment for CAR-T therapies to take on the effect (31, 72, 73). Mechanisms of aforementioned strategies need to be completely understood before any other novel strategies can be brought up including, e.g., how T cells are activated by CARs and how each component of CARs is optimized to enable efficient targeting and killing of CAR-T cells against solid tumors.

Extensive computational efforts have been devoted to explore molecular features of T cells, B cells, NK cells or any of their combinations, in tumors or tumor microenvironment for the prognosis of the immunotherapeutic response of a solid tumor (35, 74, 75), which not only helps clinicians make correct decision on whether an immunotherapy or which therapeutic modality is feasible to give, but also contributes in identifying the molecular driving force leading to immunotherapeutic resistance towards improved drugging strategies.

#### 2.2.2 Differential Efficacies of Co-Stimulatory Molecules

The patients’ endogenous immunity needs to be suppressed pre-CAR-T treatment to allow the persistence and expansion of infused T cells (76). Patients thus suffer from the adverse effects of adjunctive treatments such as chemo- and/or radio- therapies, rendering the outcome of the standard modality double-edged, i.e., improved efficacy is in sacrifice of patients’ immunity. The addition of co-stimulatory domains may take over the role of adjunctive therapies by empowering CAR-T cells to proliferate and expand. Differential use of co-stimulatory molecules is needed for different types of cancers. For example, coupling CD20 with CD137 and CD3ζ can cause prolonged tumor regression for advanced diffuse large B cell lymphomas (39); autologous or donor-derived T cells expressing a CAR that targets CD19 and harbors CD137 and the CD3ζ moiety can cause regression of extramedullary B-cell lineage acute lymphocytic leukemia (37). It was demonstrated that CD137 is a more effective costimulatory domain of CD19 CAR-T cells than CD28 regarding therapeutic persistency in clinical trials (38). Thus, balancing components of the immune system to reach the desired clinical outcome, as featured by CAR-T therapies, represents a new way of conceptualizing dosing in medicine and needs to be optimized, on a case-by-case basis, towards each type of malignancy or even per patient to achieve the best response.

### 2.3 Over-Inefficiency

#### 2.3.1 Cytokine Storm and Tumor Lysis Syndrome

CAR-T is advantageous in the relatively short response time it takes to show effects (often in days to weeks). However, it is dangerous to trigger such a dramatic immunogenic response that implies elevated potential for the occurrence of uncontrollable or even lethal side effects. For instance, the British company TeGenero almost killed six volunteers in a phase I study testing the efficacy of TGN1412 (an anti-CD28 monoclonal antibody) in the treatment of B-cell tumors and autoimmune disorders due to the induction of toxic levels of cytokines *in vivo*, referred to as the cytokine storm (77). The CAR-T associated cytokine storm can occur within the first few days after T cell infusion characterized by high serum cytokine levels, fever, vascular leakage, hypotension and even death (78). Norelli et al. simulated CAR-T associated cytokine storm using humanized mice, in which the endogenous immune system was replaced with human immune cells, thereby avoiding the induction of graft-versus-host disease after human CAR T cell infusion (41). Such an immune avalanche can also lead to the tumor lysis syndrome, which occurs when many tumor cells die rapidly following the release of cell contents into the blood and is caused by the infiltration of a large amount of lysed components of dead tumor cells into the blood. The safety issues linked to CAR-T have now drawn considerable attention. Several strategies have been proposed to control the side effects and launched with preclinical success. For example, suicide genes can be designed to easily ablate CAR-T cells and activated on abnormal CAR-T cell persistence or under acute toxicity, and such a technology is called ‘suicide gene design’ (79). Inducible Caspase 9, namely iCasp9, is a well-studied suicide gene for CAR-T therapies (40, 80). It consists of iCasp9 (the intracellular domain of the human Caspase 9 protein) that functions in inducing cell apoptosis and a human FK506 binding protein that dimerizes on the presence of small molecules such as AP1903 (81). Activation of iCasp9 in patients transplanted with CAR-T cells could rapidly induce the T cell apoptosis to cease the cytokine storm (82). Effective elimination of T cells has been observed in the pre-clinical studies on CAR-T cells carrying the iCasp9 design (83); joint use of iCasp9 and anti-GD2 CAR-T cells have been administrated in clinics, with one being completed in US (NCT02107963) and the other under recruitment in China (NCT02992210). Aside from these clinical successes, novel design on suicide genes and endeavors on other strategies as well as standardized clinical practice in validating the efficacy of CAR-T therapies await to be established.

#### 2.3.2 Myelosuppression and Severe Hepatotoxicity

The CAR-T cells can persist for years with the potential of preventing cancer relapse. This, however, may lead to serious safety concerns including myelosuppression and severe hepatotoxicity (44). Han W. D. et al. reported in 2015 their observation of a remarkable decrease followed by a gradual augmentation of blasts in the bone marrow after administrating autologous CART-33 cells to an acute myeloid leukemia (AML) patient (45). These warrant further efforts to personalize the effective drug duration of CAR-T products according to, e.g., the genetic profile and pathological state of each patient.

### 2.4 Difficulties in Product Generalization Without Sacrificing Product Efficacy

Product comparability imposes a great concern towards the large-scale application of CAR-T therapies. It is challenging to clearly define the dosage, design and calculation method of infused CAR-T cells that considerably affect the efficacy of CAR-T therapies. The total number of CAR-T cells infused varies from 108 to 1010, in clinical practice, according to the body surface area and weight of the patient. It is also possible to determine the amount of infused CAR-T cells by the CAR-positive cohort. How to reach consensus on the requirements of these detailed specifications to standardize the use of CAR-T while taking into account the heterogeneous nature of CAR-T therapy remains challenging. Some groups have been exploring the feasibility of using third party donor gene modified T cells for treating viral infections (46, 47), with the hope of creating a universal cell therapy. For CAR-T therapy, fully compatible cell bank, low immunogenic umbilical cord blood cells, allogeneic natural killer cells, and gene-editing T cells have been considered as the off-the-shelf sources for the sake of uniformity and safety (84, 85).

## 3 When Onco-immunotherapy Meets Cold Atmospheric Plasma

### 3.1 Cold Atmospheric Plasma

Cold atmospheric plasma (CAP) is an emerging onco-therapeutic tool (32). It generates a collection of reactive oxygen and nitrogen species (RONS) such as hydrogen peroxide (H_2_O_2_), ozone (O_3_), singlet oxygen (O), hydroxyl radical (OH·), superoxide (O^2−^), nitric oxide (NO·), anionic (OONO^−^) and protonated (ONOOH) forms of peroxynitrite. The cocktail of CAP is comprised of long-lived species such as H_2_O_2_ and short-lived species such as O. While long-lived species function inside cells to induce apoptosis or necrosis *via* imposing oxidative/nitrosative stress to cells, short-lived species could induce immunogenic cell death that kill cancer cells located at the long distance (86). Both long- and short-lived species function together to trigger selective death of cancer cells. The selectivity of CAP on cancer cells is achieved *via* interactions between various components and malignant cells. Malignant cells typically have a high local concentration of catalysis on the surface that prevent H_2_O_2_ entry; H_2_O_2_ and peroxynitrite interact to generate O and amplify its signaling that ultimately leads to H_2_O_2_ influx; H_2_O_2_, once entering cells, modulate intracellular redox level to halt cells at a certain cell cycle stage, trigger apoptosis or necrosis depending on the intracellular redox level after CAP exposure (87).

The selectivity of CAP against cancer cells has been demonstrated in several types of malignancies including e.g. melanoma (88), pancreatic cancer (89), and breast cancer (90). In clinics, the first clinical trial testing the efficacy of CAP in being used as an oncotherapy has been issued in July 2019. Though many studies have reported the use of CAP in rewiring the resistance of cancer cells towards chemotherapies (91, 92), synergies between CAP and drugs such as chemotherapy or immunotherapy have not been tested or launched in clinics. Below, we forecast and discuss the potential aid of CAP in enhancing the efficacy of immunotherapies and preventing its possible side effects.

### 3.2 CAP May Enhance CAR Efficiency by Boosting Cancer Cells’ Sensitivity to Immunotherapies

**Figure 3:**
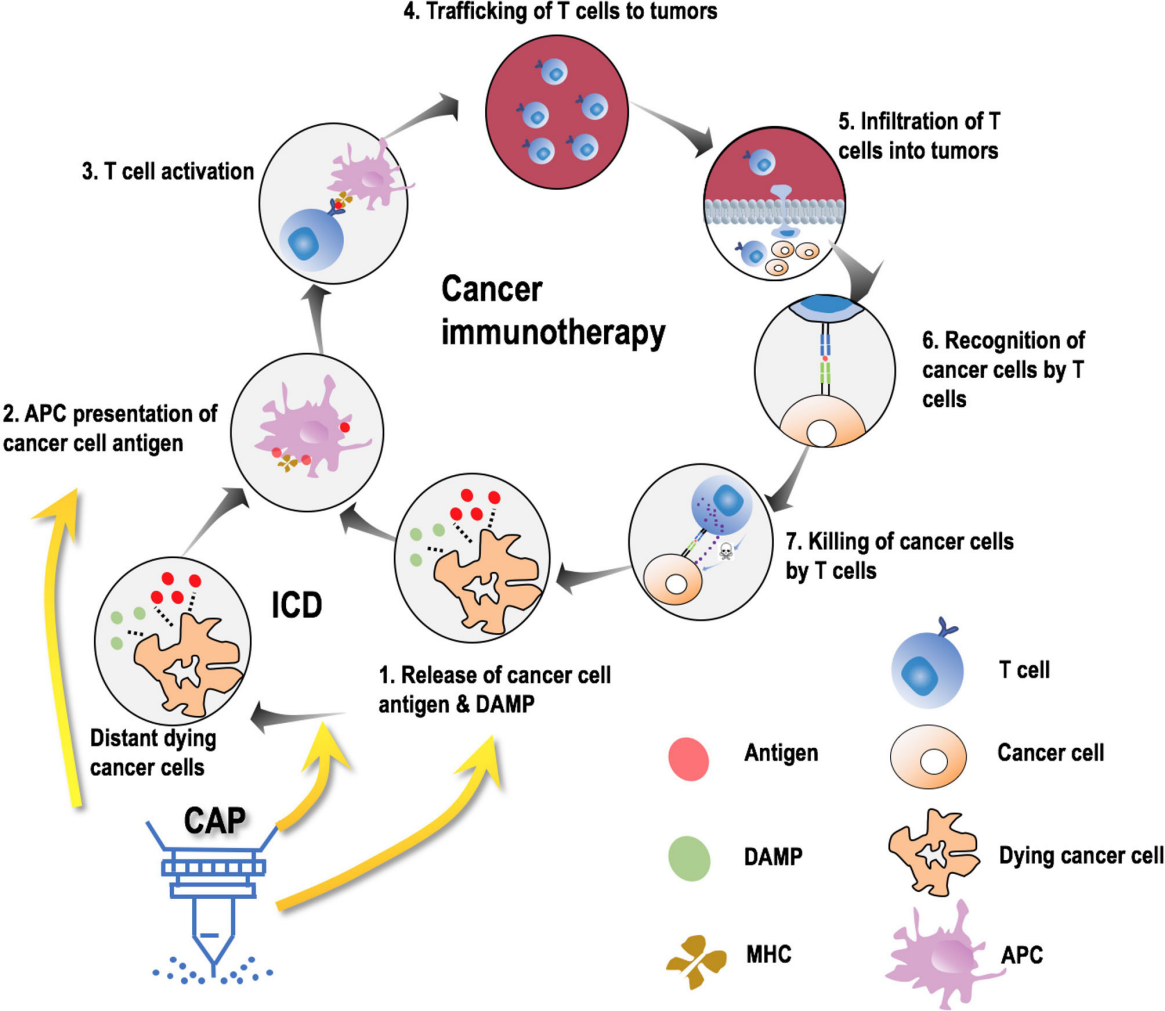
Possible molecular mechanisms leading to synergies between CAP and onco-immunotherapies. Cancer immunotherapy is comprised of 7 steps, i.e., release of cancer cell antigens and damage associated molecular patterns (DAMPs), antigen presentation cell (APC) presentation of cancer cell antigens through major histocompatibility molecules (MHC), T cell activation, trafficking of T cells to the site of tumors, infiltration of T cells to tumors, recognition of cancer cells by T cells, and killing of cancer cells by T cells. Cold atmospheric plasma (CAP) avails in this process by primarily contributing to the first two steps in three aspects, i.e., promoting immunogenic cell death (ICD), promoting MHC expression, facilitating DAMP release.

The multimodality nature of CAP could selectively kill cancer cells by breaking their more vulnerable redox equilibrium as compared with normal cells (90), induce immunogenic cell death (ICD) by increasing the visibility of malignant cells to immune cells (86, 93), and edit tumor microenvironment (TME) towards a more immune-sensitive environment by switching M2 macrophages (immunosuppressive) to the M1 state (pro-inflammatory) (94, 95), suggesting its potential in creating synergies with CAR-T cells in treating solid tumors. During cancer immunotherapy, cancer cells release antigens that are presented by antigen-presenting cells (APCs) followed by activation of T cells that infiltrate tumors and kill cancer cells (96). ICD, inducible by oxidative stress and capable of triggering the adaptive immune response, can transform non-immunogenic cells to immunogenic cells towards enhanced antigenic substance release that promotes anti-tumor immunity (96). Accumulating *in vitro* and *in vivo* evidences have demonstrated the efficacy of CAP in inducing ICD in many cancers such as colorectal tumors, pancreatic cancers, glioblastoma, and melanoma (93, 97–101), suggestive of the role of CAP in sensitizing tumors to immunotherapies (**Figure 3**). In addition, we found previously that CAP could selectively kill triple negative breast cancer cells, and this type of breast cancers is featured by high cancer stemness (90), implicating the functionality of CAP in targeting cancer stem cells; and such a property can be used to rewire lineage switch caused by CAR-T cells against CD19 for prolonged therapeutic efficacy and reduced recurrence rate. Importantly, CAP could be administrated in the form of liquids (102), thus the reactive species it delivers could more easily infiltrate solid tumors than engineered T cells due to their much smaller size, boosting CAP’s role in assisting CAR-T therapies for improved efficacy delivery.

### 3.3 CAP May Avoid Immune Over-Reaction by Reducing the Dose of Immunotherapies

The aforementioned side effects caused by uncontrolled over-efficiency of CAR-T cells (either *via* fast response or long-lasting effect) may be tempered by taking the joint use of CAP and immunotherapies by creating synergies. ROS-inducing therapies have been shown capable of stimulating the immune system and sensitizing resistant cancers to chemo- and immunotherapies (103, 104). Mechanistically, ROS could facilitate the release of damage-associated molecular patterns (DAMPs) into TME that lead to activated macrophages and enhanced antigen presentation, and promote the expression of major histocompatibility complex (MHC) I (105) that counteracts the intratumoral downregulation of CD8+ T cell response towards improved tumor antigen presentation in TME (**Figure 3**). Thus, CAP, relying on ROS to take on actions, may reduce the amount and frequency of CAR-T cells infused to the patient to achieve the desired therapeutic efficacy, and thus reduce the probability of causing side effects related to over-activated immune response. On the other hand, CAP is a mild approach with multiple clinical applications, the safety of which has been rigorously examined and clinically validated for years (106–111). Being a treatment strategy with multi-modality nature, CAP has demonstrated its power in creating synergies with chemotherapies such as Temozolomide (91) and Bortezomib (92), as well as rewiring drug resistant cells to a chemo-sensitive state (112). It is thus worthwhile to investigate the potential synergies created between CAP and CAR-T therapies for improved efficacy and reduced side effects.

## 4 Discussion

ACT immunotherapies have demonstrated tremendous efficacies in the control of complex diseases such as cancer. Among them, CAR-T therapies have considerably enriched the current oncotherapeutic modalities and revolutionized our conception in treatment and dosing given their extreme heterogeneity and flexibility by design. This, on one hand, provides us with opportunities for curing highly variable complex diseases and taking personalized medicine to an extreme and, on the other hand, imposes us tremendous challenges regarding appropriate harness on such therapeutic strategies and their large-scale production as well as applications.

The promise delivered by CAR-T therapies is tempered by the safety concerns, which primarily include ‘off-tumor’ toxicity, cytokine storm and tumor lysis syndrome, myelosuppression and severe hepatotoxicity. Besides concerns relevant to CAR design, safety issues may also arise from inappropriate manufacturing. For instance, due to an unintentional introduction of the CAR gene into one single leukemic B cell during T cell manufacturing, the mistakenly engineered product bound in cis to the CD19 epitope of leukemic cells and masked them from being recognized by anti-CD19 CAR, leading to patient relapse and CTL019 resistance (113). Thus, despite the ongoing efforts paid to improve our control on CAR-T therapies, special care needs to be paid to the manufacturing process and quality of CAR-T cells. These, collectively, require the establishment of novel strategies and technologies through joint efforts from biologists, computational scientists, clinicians and technicians.

**Figure 4:**
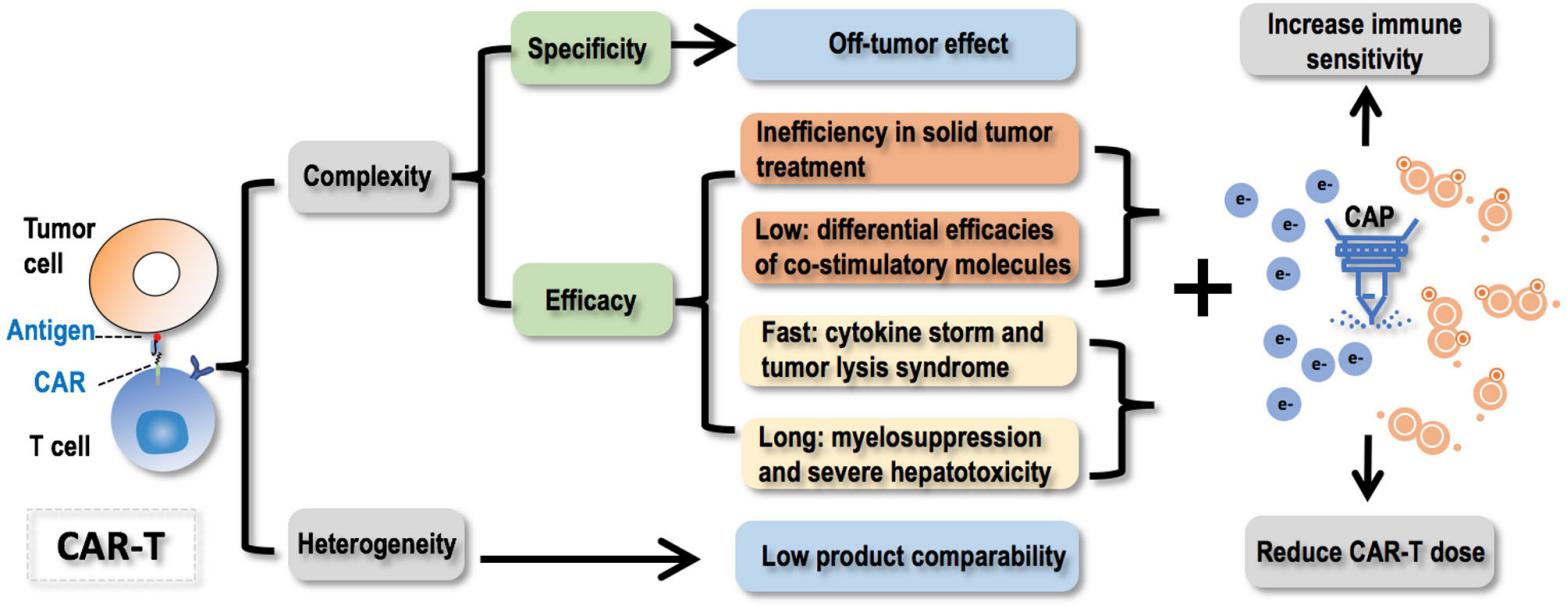
Conceptual scheme illustrating challenges faced by CAR-T therapies, opportunities CAP provided to resolve these problems, and the ultimate goal towards the complete harness of CAR-T cells as a key element of the next generation of precision medicine. CAR-T therapies are difficult to control and heterogeneous in nature. Regarding the ‘uncontrollability’, failure in controlling the specificity will lead to the off-tumor effect; failure in controlling the efficacy can be casted from 4 aspects that fall into two categories, i.e., ‘inefficiency’ including ‘inefficiency in solid tumor treatment’ and ‘differential efficacies of co-stimulatory molecules’, ‘over-efficiency’ including ‘cytokine storm and tumor lysis syndrome due to too fast therapeutic response’ and ‘myelosuppression and severe hepatotoxicity’ due to long-lasting treatment efficacy. Regarding the ‘heterogeneity’ feature, it will lead to low product comparability. CAP (cold atmospheric plasma) offers opportunities solving these challenges by increasing the immune sensitivity of cancer cells and reducing the dose of CAR-T to achieve improved or comparable efficacy with reduced recurrence and increased safety. Faced by these problems, joint efforts from biologists, computational scientists, and clinicians are needed to collaboratively advance the strategies and technologies to improve our controllability on the specificity and efficacy of CAR-T therapies, as well as find a balance between the personalization and generalization of such promising approaches.

How to deliver the desirable efficacy constitutes another major concern limiting the wide applications of CAR-T therapies. These mainly include inefficiency in treating solid tumors, differential efficacies of co-stimulatory molecules, and difficulties in product generalization without sacrificing or compromising product efficacy. Despite the ongoing effort and conventional approaches in solving these issues, we need to keep aware of emerging oncotherapeutic tools and their potential in enhancing the efficacies of CAR-T therapies. For example, CAP, whose selectivity against cancer cells and multi-modality nature may enable it an ideal adjuvant therapy or jointly used approach for CAR-T therapies to sensitize resistant solid tumors to immunotherapies and reduce the amount of infused CAR-T cells to both achieve the desired efficacy and solve the safety problem (**Figure 4**). Yet, how to jointly administrating CAP and CAR-T to patients such as the interval and frequency need to be carefully tested and designed to enable desirable outcome. It is also possible that T cells behave differently on CAP exposure that could be taken advantages of which, however, requires intensive investigations before any conclusion could be drawn.

CAR-T therapies could be used to reprogram T cells towards targeting tumor-specific antigens given a patient, taking oncotherapeutics to an extreme of personalization. This, however, may demand systematic genetic screen for each patient on mutations unique to tumors and impose too much challenges on its design, cost and production, largely restricting its wide application. How to find a proper balance between the personalization and generalization of such therapies to get each patient benefit from this promising revolutionary life-saving and emerging first-line therapeutic approach remains challenging.

## 5 Conclusion

In conclusion, it is crucial to find a balance between personalization and generalization towards improved controllability on the specificity and efficacy of CAR-T therapies. To be specific, CAR should be designed with care to avoid the off-tumor effect, enhance solid tumor treatment efficacy, improve product comparability, and resolve issues such as differential efficacies of co-stimulatory molecules, cytokine storm, tumor lysis syndrome, myelosuppression and severe hepatotoxicity. Importantly, we propose the potential synergies between immunotherapies and CAP, an emerging onco-therapeutic approach exploiting unique chemical and physical features of the fourth state of matter to deliver mild yet effective doses of reactive species, towards improved immunotherapeutic efficacies against cancers with reduced side effects.

## Author Contributions

XD conceived the study and drafted the manuscript. XD, YC and JL prepared the figures and tables. XD provided the financial support. All authors have read and proved the content of the manuscript.

## Funding

This work was supported by the National Natural Science Foundation of China (Grant No. 81972789, 82004397), Fundamental Research Funds for the Central Universities (Grant No. JUSRP22011), Technology Development Funding of Wuxi (Grant No. WX18IVJN017), Major Project of Science and Technology in Henan Province (Grant No. 192102310437), the Major Project of TCM research in Henan Province (Grant No. 2019ZYZD02, 2018ZYZD01). The funding bodies played no role in the design of the study and collection, analysis, and interpretation of data and in writing the manuscript.

## Conflict of Interest

XD was employed by CAPsoul Biotechnology Company, Ltd, Beijing, China.

The remaining authors declare that the research was conducted in the absence of any commercial or financial relationships that could be construed as a potential conflict of interest.

## Publisher’s Note

All claims expressed in this article are solely those of the authors and do not necessarily represent those of their affiliated organizations, or those of the publisher, the editors and the reviewers. Any product that may be evaluated in this article, or claim that may be made by its manufacturer, is not guaranteed or endorsed by the publisher.
